# OXR1 STABILIZES THE RETROMER TO EXTEND LIFESPAN AND NEURONAL HEALTH BY DIETARY RESTRICTION

**DOI:** 10.1093/geroni/igac059.2661

**Published:** 2022-12-20

**Authors:** Kenneth Wilson, Sudipta Bar, Eric Dammer, Birgit Schilling, Nicholas Seyfried, Hugo Bellen, Lisa Ellerby, Pankaj Kapahi

**Affiliations:** Buck Institute for Research on Aging, Novato, California, United States; Buck Institute for Research on Aging, Novato, California, United States; Emory University School of Medicine, Atlanta, Georgia, United States; Buck Institute for Research on Aging, Novato, California, United States; Emory University School of Medicine, Atlanta, Georgia, United States; Baylor College of Medicine, Houston, Texas, United States; Buck Institute for Research on Aging, Novato, California, United States; Buck Institute for Research on Aging, Novato, California, United States

## Abstract

Dietary restriction (DR) delays aging and neurodegeneration, but the mechanisms behind this remain unclear. We reared over 150 fully sequenced fly strains from the Drosophila Genetic Reference Panel under ad libitum feeding or diet-restricted conditions and measured lifespan as well as healthspan to identify new targets for DR-mediated longevity. Through genome-wide association study, we identified genetic variants associated with influencing these traits under each dietary condition. A variant in mustard (mtd, called Oxidation resistance 1, OXR1, in humans), significantly associated with DR-specific lifespan. We demonstrate that mtd/OXR1 in neurons is necessary for DR-mediated lifespan extension. Neuronal knockdown of mtd also accelerates sensory decline, arguing for a specific role of mtd/OXR1 in neuroprotection. We show that mtd is essential for stabilizing the retromer complex, which is necessary for trafficking transmembrane proteins and lipids for reuse. As a result of OXR1 deficiency, the retromer destabilizes and lysosomes become overused. Overexpression of retromer proteins or supplementation with chaperone compound R55 rescues the lifespan defects and neurodegeneration seen in mtd-deficient flies, and R55 is capable of rescuing lysosomal aggregation and OXR1-retromer co-localization in cells from humans with OXR1 deficiency. We further show through multi-omic analyses in flies and humans that mtd/OXR1 associates with accelerated transcriptomic aging and proteins involved in neurodegenerative diseases, including Alzheimer's disease (AD). Overexpression of OXR1 and retromer proteins rescued AD-associated phenotypes in a fly model of AD. Thus, mtd/OXR1 enhances protein recycling in response to DR through the retromer, improving neuronal health and lifespan through mechanisms conserved across species.

